# Tackling somatic DNA contamination in sperm epigenetic studies

**DOI:** 10.3389/frph.2025.1506117

**Published:** 2025-02-05

**Authors:** Anamika Kumari, Rajender Singh

**Affiliations:** ^1^ Division of Endocrinology, CSIR-Central Drug Research Institute, Lucknow, India; ^2^ Academy of Scientific and Innovative Research (AcSIR), Ghaziabad, India

**Keywords:** sperm DNA, DNA methylation, somatic cell contamination, sperm quality, epigenetics

## Abstract

**Introduction:**

Recent interest in sperm epigenetics has stemmed from its implication in sperm DNA quality, sperm fertility, environmental toxicity, and transgenerational inheritance. Sperm epigenetic data may be significantly affected by somatic DNA contamination, resulting in misleading conclusions. However, detecting and dealing with somatic DNA contamination in semen samples can be a challenging task.

**Methods:**

In the present study, we worked out a detailed and robust plan to deal with somatic cell DNA contamination in sperm epigenetic studies in order to draw error-free scientific conclusions. Apart from incorporating simple quality checks, such as microscopic examination and somatic cell lysis buffer (SCLB) treatment, we compared the Infinium Human Methylation 450K BeadChip data for sperm and blood samples to identify the CpG sites that were highly methylated in blood samples in comparison to sperm, but were unrelated to infertility.

**Results and discussion:**

The comparison of Infinium Human Methylation 450K BeadChip data for sperm and blood samples identified 9564 CpG sites that can be used as markers for analyzing somatic DNA contamination. We have put together a comprehensive plan including evaluation under a microscope, SCLB treatment, inclusion of CpG biomarkers for sample quality evaluation, and applying a 15% cut off at the time of data analysis to completely eliminate the influence of somatic DNA contamination in sperm epigenetic studies. We conclude that if this comprehensive plan is followed, the influence of somatic DNA contamination in sperm epigenetic studies can be completely eliminated.

## 1 Introduction

Sperm epigenetic analysis can have value as a significant biomarker for sperm quality analysis in assisted reproduction (1, 2). In a previous study, we postulated that oligozoospermic individuals not only present with a decreased sperm count, but they also carry significant epigenetic anomalies in their sperm DNA (2), and this could have a significant impact on embryonic development and/or the health of the next generation. Spermatogenesis is particularly designed to involve DNA methylation reprogramming, such that spermatocytes undergo demethylation followed by selective re-methylation in spermatids/sperm (3). In disturbed spermatogenesis, germ cells displayed altered DNA methylation in transposable elements and genes involved in the process of spermatogenesis (3). In addition to this, alterations in sperm DNA methylation are also of significance from other perspectives of evaluating environmental exposure and toxicity (4). Epigenetic modifications as a result of adverse environmental exposure may account for transgenerational changes (5). All the above make the investigation of sperm DNA methylation critical for understanding sperm quality, fertility, infertility, and transgenerational inheritance.

Semen samples are often contaminated with somatic cells, the chances of which increase several folds in oligozoospermic individuals (6). Therefore, it is imperative to rule out somatic cell contamination in order to clearly understand the epigenetic changes in sperm/germ cells. Microscopic examination of a semen sample can detect somatic cells/leukocytes when present in significant numbers; however, this analysis may fail to detect contamination at a lower level (7). In germ cells, the DNA methylation level of a number of genes is much lower in comparison to somatic cells (8, 9). Therefore, a few contaminating somatic cells may significantly bias the DNA methylation analysis to an interpretation of differential methylation in sperm. Treatment with somatic cell lysis buffer has been suggested, which removes the contaminating cells to a significant level; however, it can never ascertain the complete elimination of somatic cells, even after re-examination under a microscope (10).

It has been suggested that DNA methylation analysis of genomic regions highly methylated in somatic cells in comparison to germ cells can help identify somatic cell contamination in sperm samples (9, 10). However, a comprehensive plan to be followed to achieve the complete elimination of somatic cells in sperm epigenetic studies has not been put together. In the present study, we have provided a detailed experimental plan to deal with the problem of somatic DNA contamination in sperm samples. This well-defined strategy, if followed, can completely eliminate the influence of somatic cell contamination, leading to a highly accurate interpretation of epigenetic changes in sperm samples.

## 2 Methods

### 2.1 Somatic cell lysis

Fresh semen samples were firstly washed twice with 1X PBS by centrifugation at 200 g for 15 min at 4°C, followed by sample inspection under a microscope (Nikon Eclipse Ti-S Inverted microscope with 20X objective lens) to identify the level of somatic cell contamination and count the number of sperm. After washing with 1X PBS, samples were incubated with freshly prepared somatic cell lysis buffer (SCLB) (0.1% SDS, 0.5% Triton X-100 in ddH_2_O) for 30 min at 4°C. Samples were again checked under a microscope to detect the presence of somatic cells and sperm count was performed again. If any somatic cell was detected, the samples were centrifuged to obtain the pellet, and SCLB treatment was repeated. If no somatic cells were detected, sperm were pelleted by centrifugation, followed by PBS wash to obtain the highly pure sperm population.

### 2.2 450 K array comparison between sperm and blood cells

In our previous study on human sperm, we used the Infinium Human Methylation 450K BeadChip (Illumina) to analyze genome-wide methylation in 12 blood samples (6 normozoospermic fertile and 6 oligozoospermic infertile) (1). Similarly, in another study, we analyzed the same set of markers in 12 sperm samples (6 normozoospermic fertile and 6 oligozoospermic infertile) (2). This platform analyzes 482,421 CpG sites and 65 random single-nucleotide polymorphisms covering 21,231 RefSeq genes for the genome-wide methylation scan (1, 2). These studies involved comparisons of DNA methylation in the cases with controls to identify differentially methylated regions in sperm and blood DNA of infertile individuals. In addition to identifying differentially methylated regions linked with infertility, these studies also generated data for the comparison of blood and sperm DNA methylation across a number of regions spread over the entire human genome. To identify the spots specifically with high methylation in somatic cells and low methylation in sperm, we compared the microarray data for both blood and sperm to identify CpG sites with high methylation (>80%) in blood and low methylation (<20%) in sperm from the set of CpGs that were not differentially methylated in infertility. Such CpG sites can be used for the identification of somatic cell contamination in sperm preparation for epigenetic studies.

### 2.3 The possibility of proxy methylation due to somatic cells

In healthy normozoospermic fertile men, somatic cells may be present up to 1 × 10^6^ cells/ml of semen (11), but in the case of oligozoospermic infertile men, contaminating somatic cells may be present in a much higher concentration. Therefore, somatic DNA contamination in sperm preparation may depend on a variety of factors, and samples with low sperm count are particularly vulnerable to high contamination (6). Further, with decreasing sperm count, the influence of a small percent of somatic cells in the sample is going to be more significant. Somatic and germ cells methylomes are completely different, as the majority of the promoters in sperm are hypomethylated (12). However, various biological or environmental factors may affect the methylation status of some of these regions and account for infertility or developmental disorders (1, 2). Since such hypermethylation may also occur due to somatic cell contamination rather than actual alteration in sperm DNA, it is essential to distinguish sperm DNA differential methylation from a proxy methylation signal coming from contaminating somatic cells.

### 2.4 Calculations for the cut off value

**Figure 1:**
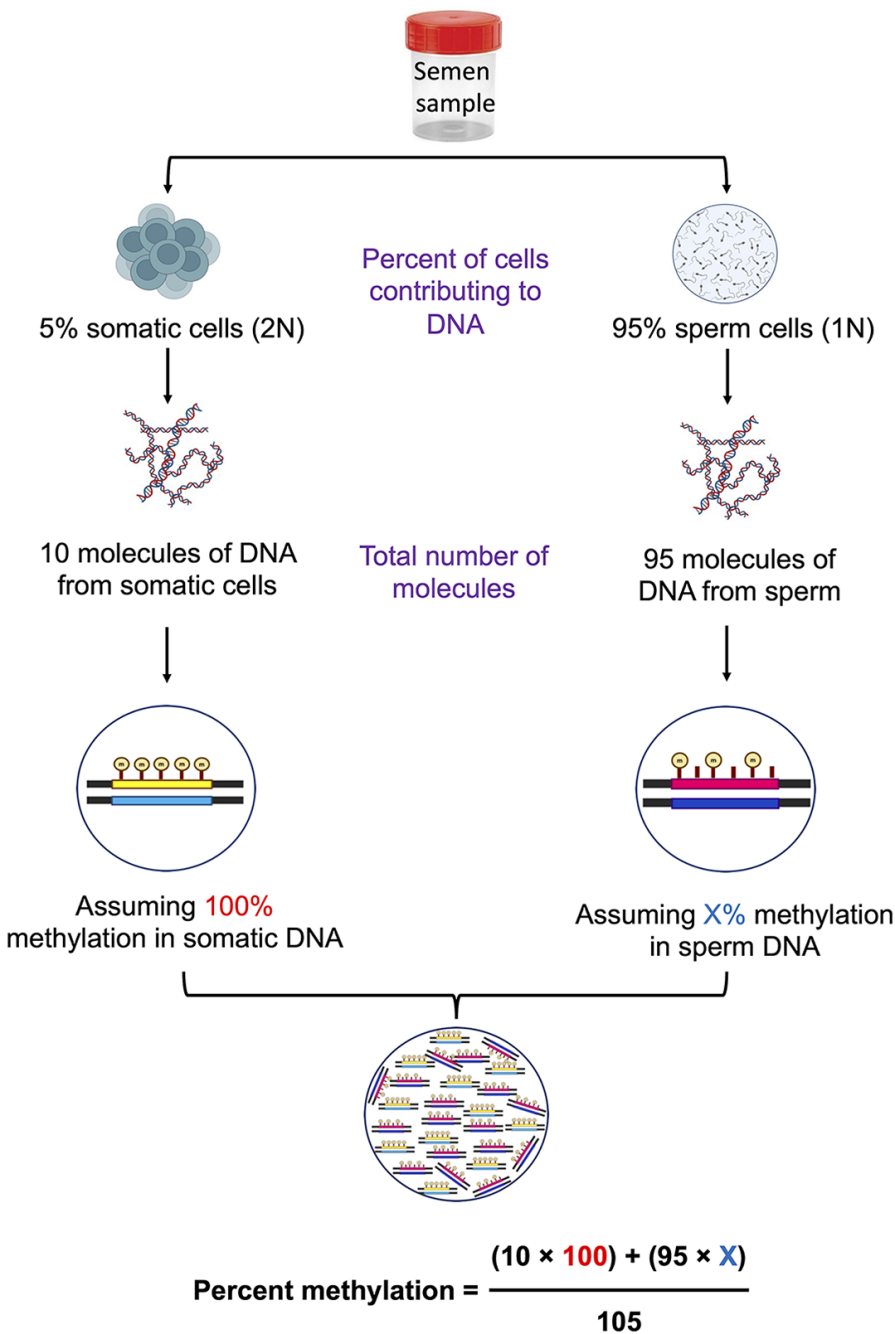
Schematic diagram showing the calculations of percent methylation in the presence of contaminating somatic cells.

Somatic cells in a semen sample can be visualized under a microscope to find the level of contamination; however, if the somatic cell contamination is below 5% of sperm number, confirming their presence becomes challenging. There are possibilities that the complete elimination of somatic cells may never be achieved even after following multiple precautionary steps, such as swim-up/density gradient and SCLB treatment. Considering this possibility, we thought of introducing another checkpoint at the level of data analysis. We performed overall DNA methylation calculations assuming various combinations of case and control samples with the base assumption of an undetectable level of 5% somatic cell contamination (Figure 1). Methylation percentages were calculated considering two situations, i.e., total overall methylation level in sperm samples with and without somatic contamination. Inverse scenarios of DNA methylation level between cases and controls were considered to arrive at the most contrasting scenario that could affect the overall methylation percentage calculations and the ultimate inference. In the end, differential methylation was calculated considering four different situations as follows: (1) both control and case samples are contaminated, (2) the case sample is contaminated and the control is contamination free, (3) the case is not contaminated and the control is contaminated, and (4) both case and control are contamination free. The above scenarios were used to arrive at a percentage cut-off to be applied in differential methylation data analysis.

## 3 Results

### 3.1 Somatic cell lysis removes contamination significantly

**Figure 2:**
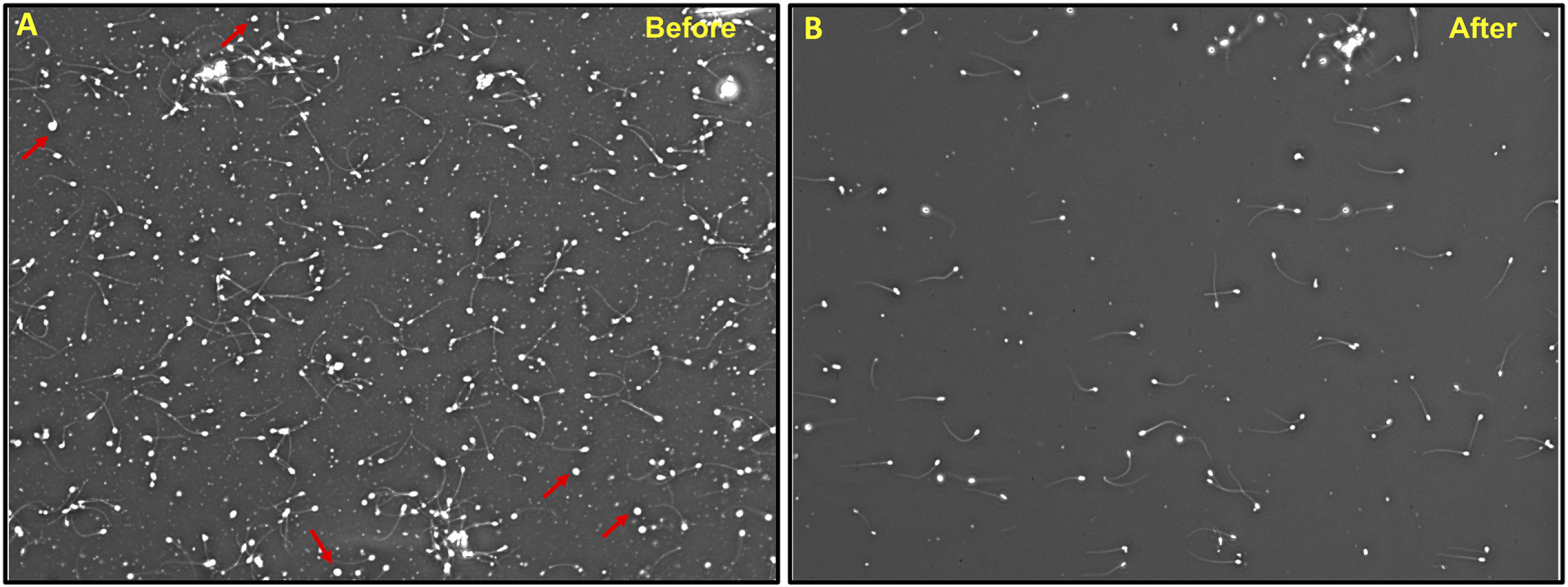
Treatment of semen sample with SCLB; **(A)** A sample before SCLB treatment showing somatic cell contamination, **(B)** the same sample after SCLB treatment showing the absence of somatic cells. WBCs are shown by arrowheads. The image is taken at 100X.

To assess the effectiveness of SCLB in removing somatic cells from sperm samples, we conducted a series of tests comparing the samples before and after SCLB treatment. Our objective was to reduce the number of somatic cells, especially leukocytes, as they can compromise the accuracy of sperm-specific epigenetic analysis. For this purpose, we selected samples with somatic cell contamination to various degrees. Prior to lysis, somatic cells were detected in sperm samples (Figure 2A). Post-lysis, microscopy revealed a significant reduction or almost complete elimination of somatic cells (Figure 2B). Treatment of semen samples with SCLB has been previously reported to be very efficient in eliminating leukocytes (13). Considering the possibility of a very low level of contamination (maximum 5%), we performed further checks to tackle the issue of hidden contamination.

### 3.2 Methylation spots for the assessment of somatic cell contamination

We considered all methylation spots analyzed in sperm and blood DNA from control samples analyzed in our previous studies (1, 2). Since the issue pertains to observing hypermethylation in sperm as a result of somatic cell contamination, we looked for CpG spots that were highly methylated in blood cells and minimally methylated in sperm, but were not differentially methylated in infertile samples in either of the two studies (1, 2). Therefore, we selected to apply a filter of more than 80% for methylation in blood simultaneously with less than 20% for methylation in sperm, and obtained 9,564 unique CpG sites that could be used as markers for analyzing somatic DNA contamination (Supplementary data sheet 1). These markers, if included in DNA methylation studies, can help identify the presence of contaminating somatic cells in sperm samples, ensuring the accuracy of downstream analyses (Supplementary data sheet 1). When working with the whole genome methylation sequencing or microarray, any of these markers can be used to assess the presence of somatic cell contamination. For a ready reference to investigators, some of the potential markers for the assessment of somatic cell contamination from this list are presented in Table 1 and a detailed description of these is available in Supplementary Table S1. DNA methylation of more than 20% across all these markers should be taken to suggest somatic contamination, leading to the exclusion of such a sample from further investigation. 

**Table 1:** CpG sites for use as biomarkers to identify somatic cell contamination by DNA sequencing methods. The corresponding genomic positions are provided in Supplementary Table S1.

CG IDs from microarray	Gene	Primers for amplification
cg21366200 cg05778559	DAZL	FP: TATTTTGCGGAGTTACGGGGAGA RP: CCTACCTAAACGCACCACAACCA Product length: 362
cg04463551 cg02899723 cg15190754 cg10585263 cg22696214 cg27311866	DDX4	FP: GATGGGTAAAAGGGGAGAGAGTAT RP: CTCCGCGACTTACTCTCCCAAA Product length: 475
cg10668096 cg02985694 cg08374687 cg09682129	ADAD1	FP: GGATAGTAAGGGAGGAGGTTGAA RP: ACCCACCAAAATTATCCTTCCT Product length: 464
cg00733190 cg12771165	STRA8	FP: ATTTTGCGAGGTGAGTTAGT RP: TCACCTATTAAACTCCGCTACAC Product length: 393

### 3.3 PCR test for assessing somatic cell contamination

**Figure 3:**
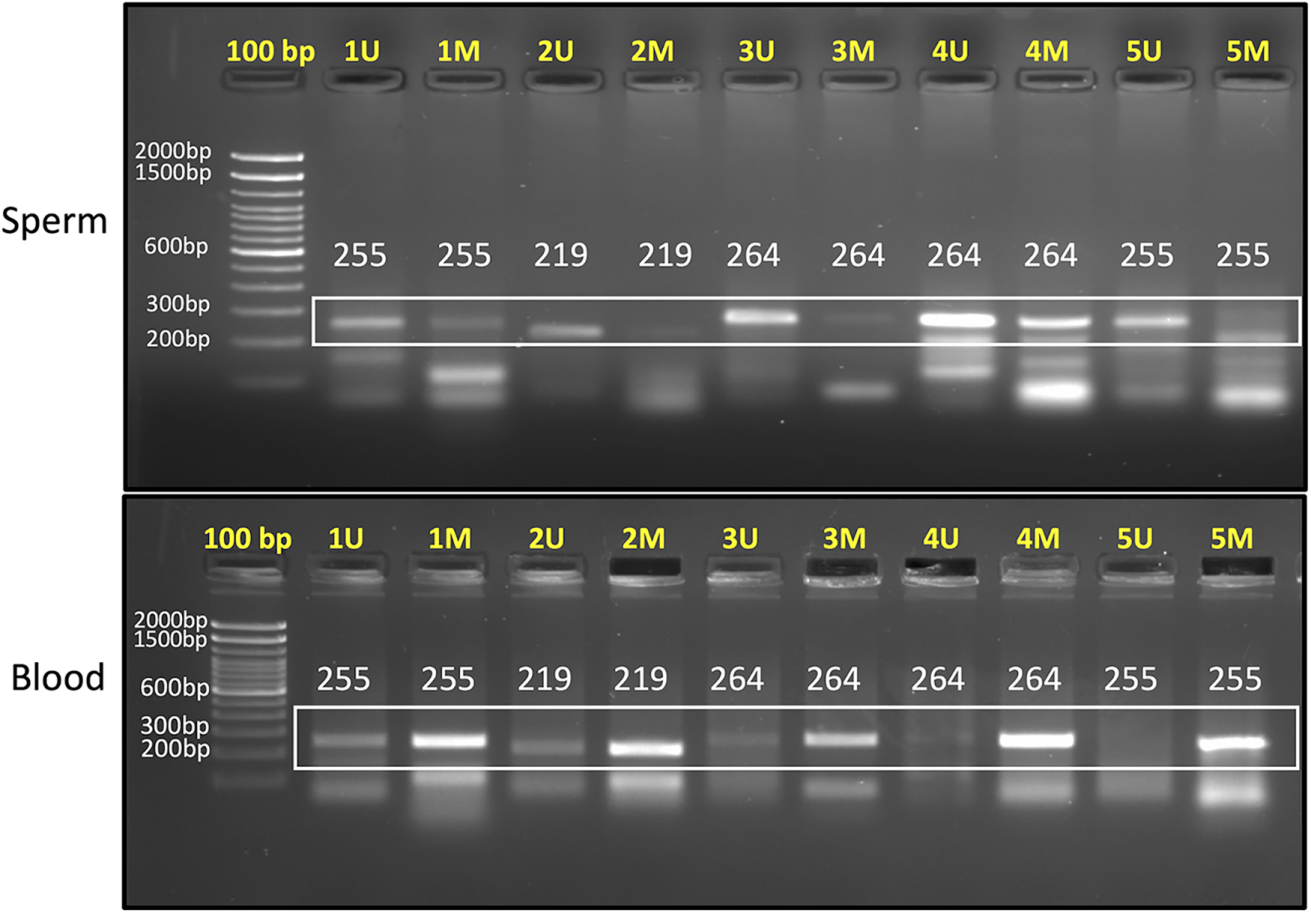
2% agarose gel image showing PCR products for sperm (upper panel) and blood samples (lower panel). “U” denotes unmethylated-specific primer, while “M” represents methylated-specific primer. The targets amplified in this gel are as follows: Primer 1 (cg09439260), Primer 2 (cg15180637), Primer 3 (cg22703164), Primer 4 (cg10668096), and Primer 5 (cg00733190). In sperm sample, unmethylated-specific primers show prominent bands and in blood samples, methylated-specific primers show prominent bands. Expected product size is mentioned in each lane. Annealing temperature for each primer set is provided in Table 2.

In resource limiting conditions, PCR is still employed to study DNA methylation. Such analysis relies on analyzing DNA methylation by methylation-specific PCR. We have also designed primers for inclusion of CpG biomarkers in PCR-based analysis of DNA methylation. For a ready reference to investigators, we have provided the methylation-specific primer sequences for CpG sites in the DAZL (cg09439260, cg15180637 and cg22703164), DDX4 (cg15190754, cg10585263 and cg22696214), ADAD1 (cg10668096 and cg02985694), and STRA8 (cg00733190 and cg07131943) genes that can be employed for the assessment of somatic cell contamination (Table 2). Out of these, we have also undertaken PCR standardization of five marker CpGs (cg09439260, cg15180637, cg22703164, cg10668096 and cg00733190) for ready inclusion in PCR-based methylation assessments. PCR reaction included 1 µl each of forward and reverse primers from 10 µM stock solution and 2 µl of nuclease free water in a PCR-tube. The above prepared reaction mixture was incubated at 70°C for 2 min and immediately after the incubation, 30 ng (1 µl) bisulfite converted template DNA and 5 µl PCR master mix (2X) were added. After this, PCR reaction was carried out using the following conditions; heating at 95°C for 1 min, followed by 35 cycles of PCR including denaturation at 95°C for 30 s, annealing at specified temperature (Table 2) for 30 s, and polymerization at 72°C for 20 s, and a final polymerization step at 72°C for 10 min. After PCR, 5ul of the PCR product was checked on a 2% agarose gel (Figure 3). The detailed description of these primers and their target sequences are provided in Supplementary Table S2. For samples free of contamination, unmethylated-specific primers show prominent band in sperm DNA, but methylated-specific primers show equally prominent or more intense band in blood samples. A blood like amplification across all primers would suggest significant contamination with somatic DNA (Figure 3). Methylation-specific PCR does not offer primers' choice; therefore, some non-specific amplification might be encountered, which is acceptable as long as the specific band is prominent. 

**Table 2:** CpG sites for use as biomarkers to identify somatic cell contamination by PCR. The corresponding genomic positions are provided in Supplementary Table S2. The first five markers were standardized for PCR amplification and the corresponding gel image is shown in Figure 3.

CpG IDs of microarray	Methylated-specific primers	Unmethylated-specific primers
cg09439260	FP: TTAGAGTTGTATTTTGTGGTGGCG RP: CTTACTAATAACGACTCCCTCGTAT Product length: 255, annealing temp 62°C	FP: TTAGAGTTGTATTTTGTGGTGGTG RP: CTTACTAATAACAACTCCCTCATAT Product length: 255, annealing temp 62°C
cg15180637	FP: GCGAGGGGATTAGAGGTATTTTCG RP: TCTCCCCGTAACTCCGCAAAATA Product length: 219, annealing temp 62°C	FP: GTGAGGGGATTAGAGGTATTTTTG RP: TCTCCCCATAACTCCACAAAATA Product length: 219, annealing temp 60°C
cg22703164	FP: TTTAGGTTTTATAGGAAGGCG RP: CTCACGTTATAAAAATCCACCGT Product length: 264, annealing temp 60°C	FP: TTTAGGTTTTATAGGAAGGTG RP: CTCACATTATAAAAATCCACCAT Product length: 219, annealing temp 60°C
cg10668096	FP: AGGGAGGAGGTTGAATTGCG RP: CCCTACAAAACCCGAACTTAC Product length: 264, annealing temp 60°C	FP: AGGGAGGAGGTTGAATTGTG RP: CCCTACAAAACCCAAACTTAC Product length:264, annealing temp 64°C
cg00733190	FP: GCGGTTAGGGATAGGGTCG RP: TCACCTATTAAACTCCGCTACAC Product length: 255, annealing temp 60°C	FP: GTGGTTAGGGATAGGGTTG RP: TCACCTATTAAACTCCACTACAC Product length: 255, annealing temp 64°C
cg15190754	FP: GGTTATTTGGTTATGAGGTTAGAGCG RP: CTCCGCGACTTACTCTCCCAAA Product length: 257	FP: GGTTATTTGGTTATGAGGTTAGAGTG RP: CTCCACAACTTACTCTCCCAAA Product length: 257
cg10585263	FP: TCGTTATAGGGGTTCGAACG RP: CTCCGCGACTTACTCTCCCAAA Product length: 231	FP: TTGTTATAGGGGTTTGAATG RP: CTCCACAACTTACTCTCCCAAA Product length: 231
cg22696214	FP: GTAGGTTAGAAGTGGAGGCG RP: ACTCCATCCACACTTTAACC Product length: 285	FP: GTAGGTTAGAAGTGGAGGTG RP: ACTCCATCCACACTTTAACC Product length: 285
cg02985694	FP: GTAAGGCGGTTAGTTGGATTCG RP: CCCTACAAAACCCGAACTTAC Product length: 218	FP: GTAAGGTGGTTAGTTGGATTTG RP: CCCTACAAAACCCAAACTTAC Product length: 218
cg07131943	FP: GTTTTTTTAGTTTAGGCGTAGTCG RP: CAAAAACAATCGAACGCTCTCT Product length:213	FP: GTTTTTTTAGTTTAGGTGTAGTTG RP: CAAAAACAATCAAACACTCTCT Product length:213

### 3.4 Apply 15% cut off during DNA methylation data analysis

We considered various possibilities of contamination of sperm with somatic cells and various levels of methylation in these cells to arrive at the best cut-off that should be applied during methylation data analysis in order to filter out the hidden possible influence of somatic contamination on data interpretation. It is evident that DNA methylation data can be biased up to 38% level in a few combinations of contaminating cells (Table 3). In the case of highly significant methylation differences between case and control samples, the overall inference would remain the same irrespective of the influence of contamination. The challenge arises when the comparison groups have small differences from each other that could be significantly biased by the presence of contaminating somatic cells. There is a high chance of having around 5% hidden somatic cell contamination in samples, which could lead to proxy methylation differences, rather than reflecting true methylation differences in sperm samples. For example, in the highlighted rows in Table 3, the difference between the comparison groups could be significantly affected by contaminating cells. Assuming that somatic cells would always be present in a sample to a certain level, we propose applying a cut off during differential methylation data analysis to get rid of the influence of the hidden contamination on the overall inference. We considered applying 5%, 10%, 15%, and 20% methylation difference cut off here. The minimum 5% cut off offers no help as the values in the highlighted rows remain above this value, 10% cut off takes care of the influence of contamination in certain situations but not in all, applying a cut off of 15% takes care of all possible situations of contamination and comparison groups, and raising the cut off to 20% serves no further purpose (Table 3), instead it reduces the number of differentially methylated regions (DMRs) highly significantly, leaving the investigators with very limited number of DMRs to interrogate further. Therefore, we settle with a cut off value of 15% differential methylation for downstream data analysis. This threshold is biologically relevant and reduces the risk of misinterpretation caused by small amounts of contamination that may not be visible under a microscope.

**Table 3:** The summary of calculations under various combinations of sample comparisons for differential methylation analysis highlighting the relevance of a 15% cut-off.

Control sample		Control methylation level		Case sample		Case methylation level		Difference			
Soma methylation level (5% cell)	Sperm methylation level (95% cell)	Total methylation without contamination (%)	Total methylation with contamination (%)	Soma methylation level (5% cell)	Sperm methylation level (95% cell)	Total methylation without contamination (%)	Total methylation with contamination (%)	Contamination -Contamination	Contamination – Pure (Case-Control)	Pure-Contamination (Case-Control)	Pure - Pure
100	100	100	100	100	10	10	18.57	−81.43	−81.43	−90	−90
100	90	90	90.95	100	20	20	27.62	−63.33	−62.38	−70.95	−70
100	80	80	81.9	100	30	30	36.66	−45.24	−43.34	−51.9	−50
100	70	70	72.86	100	40	40	45.71	−27.15	−24.29	−32.86	−30
100	60	60	63.81	100	50	50	54.76	−9.05	−5.24	−13.81	−10
100	50	50	54.76	100	60	60	63.81	9.05	13.81	5.24	10
100	40	40	45.71	100	70	70	72.86	27.15	32.86	24.29	30
100	30	30	36.66	100	80	80	81.9	45.24	51.9	43.34	50
100	20	20	27.62	100	90	90	90.95	63.33	70.95	62.38	70
100	10	10	18.57	100	100	100	100	81.43	90	81.43	90

## 4 Discussion

Recent studies on sperm epigenome have revealed the significance of DNA methylation marks in spermatozoa. Sperm DNA methylation has been shown to be a significant indicator of environmental toxicity (14, 15), exposure to endocrine disruptors (16, 17), and possible genotoxicity (18, 19). An interesting study on sperm DNA methylation over a period of 18 years (1990–2008) had shown increased DNA methylation with ageing (20), which may also explain how spermatogenesis is naturally designed to dampen with age so as to check the transmission of poor quality DNA to the next generation. Our previous studies on sperm DNA methylation identified differential DNA methylation markers that can be used as sperm fertility and quality biomarkers in assisted reproduction (2). Further, altered DNA methylation may also explain normozoospermic idiopathic infertile cases (21), which could be linked to issues with embryonic development (22, 23). In other contemporary studies, sperm DNA methylation has been implicated in transgenerational inheritance (22, 24). Given the significance of DNA methylation carried by sperm, such analyses are poised to make a significant contribution in understanding a number of biological phenomena in the future. In view of the above, it is critical to correctly identify the DNA methylation status in studies on sperm DNA.

**Figure 4:**
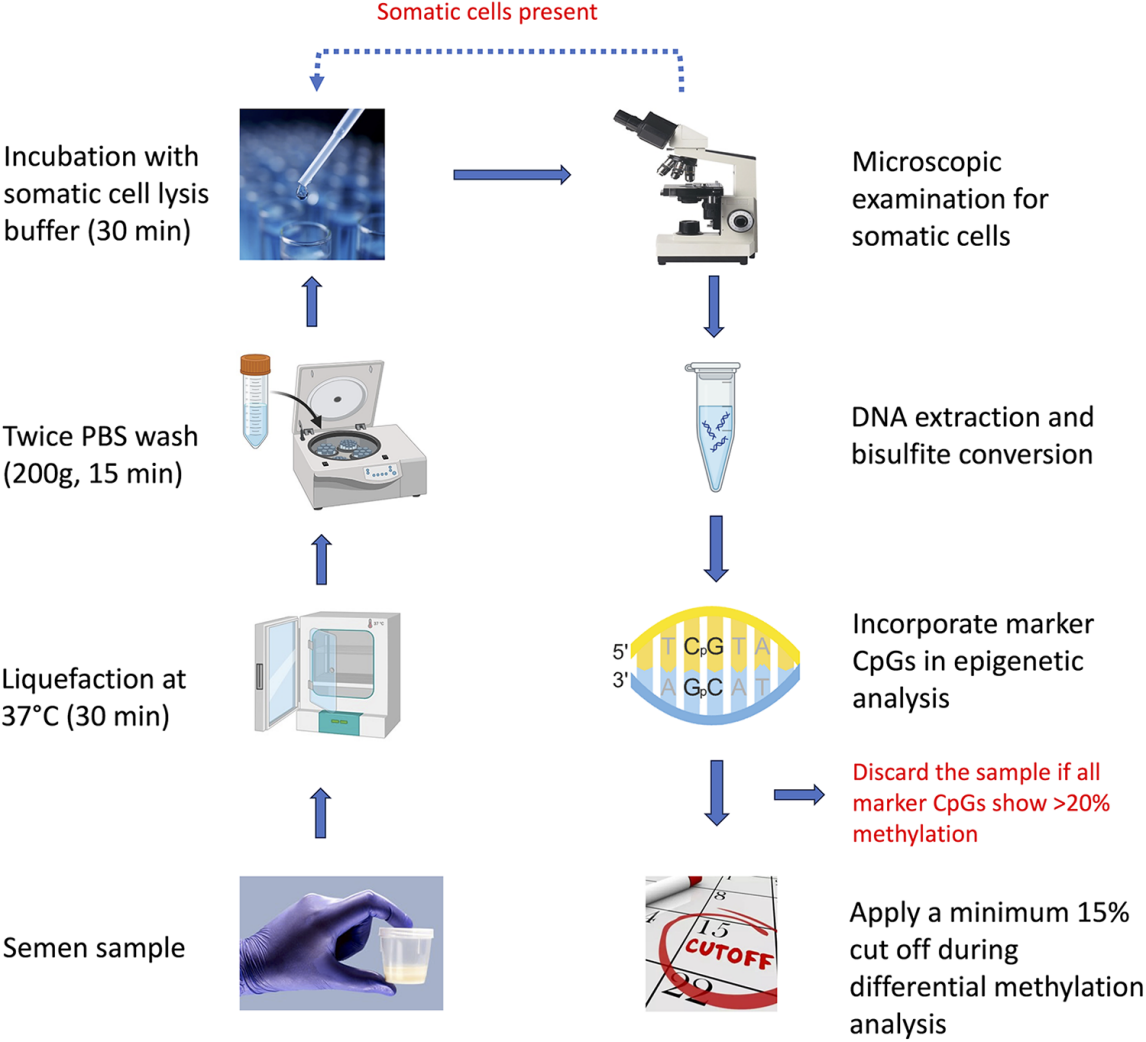
Flow-chart illustrating the overall scheme to be followed to get rid of the influence of somatic cell contamination in sperm epigenetic studies.

Somatic cell contamination or the presence of pus cells in semen, particularly in oligozoospermic infertile samples, is a common observation (25–27). Often, lower the sperm count, higher are the chances of contamination by somatic cells (28, 29). Since each somatic cell carries double the amount of DNA, they make a significant contribution to the overall DNA in a sample. We suggest following a set plan for analyzing somatic DNA contamination. The first step is sample examination under a microscope. This should detect the presence of somatic cells in a significant percentage. Treatment with SCLB should get rid of more than 95% of the somatic cells present in the sample, leaving behind the possibility of the presence of some of them, which may not be visible under a microscope or may appear miniscule in comparison to the number of sperm (Figure 4). If somatic cell contamination is still evident after following the above step, another round of SCLB treatment should be given. However, the presence of some somatic cells in a very low number cannot still be ruled out. To further detect the presence of somatic cells, we propose to include in the sequencing or PCR analysis any of the 9,564 CpGs identified in this study, which are significantly methylated in somatic cells in comparison to sperm, but are unrelated to infertility. We suggest the analysis of a minimum of three such spots in sequencing-based studies; however, depending upon the experimental capacity, the investigators may include up to 10 such spots in their analysis. More than 20% methylation across all these spots should indicate a significant contamination of the sample with somatic cells (Figure 4). However, occasional higher methylation of one to a few of these spots may be related to infertility or the trait under study. In the case of the detection of somatic cell contamination in a particular sample, it should be excluded from further data analysis.

After following the above steps, the samples should be completely free from somatic cells. Since DNA methylation studies may detect hundreds of differentially methylated regions, methylation studies generally apply a cut-off (10%–20%) to narrow down to biologically meaningful differences (30, 31). After doing a round of calculations considering all possible scenarios, we found that somatic cell contamination may make a significant impact on the interpretation of DNA methylation data when the methylation difference is in the lower range (∼10%). We considered applying 5%, 10%, 15%, and 20% cut off here. The minimum 5% cut off offers no help as the values in the highlighted rows remain above this value (Table 3), 10% cut off takes care of the influence of contamination in certain situations but not in all, and applying a cut off of 15% takes care of all possible situations of contamination and comparison groups (Figure 4, Table 3). Further, the WHO semen analysis parameters 2021 suggest that a normal semen sample should have less than 1 million WBCs (11). Taking into account 16 million per ml sperm count to be normal as per the WHO 2021 criteria, a contamination of 1 million WBCs per ml of sample would mean a maximum contamination level of 6.25% cells. Applying a cut off of 15% at the level of data analysis is able to tackle about 7% somatic cell contamination, which in itself is sufficient to tackle all somatic cell contamination in a sample classified as normal as per the WHO 2021 criteria. Investigators may choose to go for a higher cut-off to narrow down to DNA methylation differences with a higher stringency, but the 15% cut-off would also provide the investigators with enough number of DMRs for interrogating their biological meaning in the downstream analysis. Raising the cut off further to 20% does not offer any benefit, except significantly reducing the number of DMRs for further downstream investigation.

Practically, in DNA sequencing-based studies, the inclusion of a few additional target amplicons would be an ideal approach. However, in certain laboratory settings with resource constraints, it may not be possible to carry out sequencing studies, precluding the possibility of using sequencing-based methods for analyzing somatic cell contamination. Therefore, it is also important to develop and suggest a simple PCR-based method for analyzing somatic DNA contamination. The allele-specific PCR described in this study for five target regions will help in analyzing somatic cell contamination by simple PCR based assay in resource limiting conditions. In the case of PCR-based methods, we suggest incorporating a higher number of markers (at least 5) to ensure DNA quality analysis due to the possibility of PCR artifacts. The PCR-based method may have detection limits in comparison to the sequencing-based method, but would still help in getting rid of the contamination in combination with other methods suggested in this study.

We recognize certain limitations in our method, particularly regarding the use of SCLB for sperm purification. Research has highlighted issues with the use of SCLB, such as potential damage to sperm membranes and notable cell loss, often without a corresponding increase in purification quality (32). SCLB treatment affects the membrane integrity of sperm cells, leading to a significant impact on RNA and proteins, while nuclear DNA remains largely unaffected. The objective of the experiment is to isolate a sperm cell population free from contamination by somatic cells. For achieving this, a small percentage of sperm loss (33) due to the SCLB treatment can be acceptable. This loss is considered justified in the context of prioritizing the purity of the sperm population for further analysis. Nevertheless, SCLB continues to be widely used due to its practicality, affordability, and effectiveness in selectively lysing somatic cells while preserving sperm DNA for subsequent analyses. While alternative techniques like density gradient centrifugation or fluorescence-activated cell sorting (FACS) offer advantages, they are often costlier and equipment intensive. Addressing these limitations not only provides a nuanced understanding of the approach but also paves the way for future studies to optimize DNA isolation and conduct sperm epigenetic analysis. In addition to somatic cell contamination, confounding factors such as ageing, exposure to pollutants or toxins, and disease conditions including cancer or prolonged use of certain medications may introduce DNA methylation changes, which cannot be tackled by the methods proposed in this study. Therefore, in addition to tackling the issue of somatic cell contamination, it is equally important to eliminate the influence of confounding factors in sperm epigenetic studies to clearly distinguish the effect of the factor under investigation.

DNA methylation in sperm is being analyzed in the context of infertility, oxidative stress, exposure to endocrine disruptors, and transgenerational inheritance. Figuring out the germ cell epigenetic in these contexts can be critical, and somatic cell contamination may drive to misleading conclusions. Therefore, understanding the importance of and employing methods to tackle somatic DNA contamination is critical in understanding the epigenetic changes in infertility and molecular basis of transgenerational inheritance. Understanding the causes behind epigenetic changes in infertile sperm and their contribution to transgenerational inheritance may help in sperm quality analysis and infertility treatment. Sperm epigenetic markers, if identified, can help in better quality DNA transmission to the upcoming generations apart from ensuring parenthood to the current generation.

## 5 Conclusion

We conclude with putting up a comprehensive sequential plan for detecting and excluding somatic cell contamination in sperm epigenetic studies. There are quality checks at every step to ensure the removal of somatic cells or the exclusion of a sample with somatic cell contamination. In the case of a failure to detect at any of these levels and the eventual inclusion of a sample with minor contamination in the analysis, the final cut-off of 15% at the level of data analysis would ensure that somatic DNA contamination does not contribute to the overall analysis and the conclusions drawn are not biased. This way, epigenetic studies on sperm would benefit from advanced planning to ensure the complete removal of somatic DNA contamination, which may otherwise become a critical issue at the time of study evaluation during or after publication. We believe our study would provide investigators with a ready standard operating procedure to be followed in sperm epigenetic studies.

## Data Availability

The original contributions presented in the study are included in the article/Supplementary Material, further inquiries can be directed to the corresponding author.
