# Supplementary material for: Tackling somatic DNA contamination in sperm epigenetic studies
Source: Front Reprod Health. 2025 Feb 5;7:1506117. doi: 10.3389/frph.2025.1506117 (PMC11835817; doi:10.3389/frph.2025.1506117)
Supplement: Supplementary file 2 [file Table1.docx]

**Supplementary Table S1: Primer details for CpG sites used as biomarkers to detect somatic cell contamination through DNA sequencing**

| **Gene** | **CpG spot** | **Primer** | **Original sequence** |
| --- | --- | --- | --- |
| DAZL | 1. cg21366200 (GRCH38:Chr3:16604780) 2. cg05778559 (GRCH38:Chr3:16604868) | FP: TATTTTGCGGAGTTACGGGGAGA  RP: CCTACCTAAACGCACCACAACCA  Product length: 362 | GRCH38: chr3:16604725-16605086  **CATCTTGCGGAGCCACGGGGAGA**GCGCGCCAGAAATGAGGCTGGCGG  GGCGGAGGCGCGTGGGAGTGGGGGAGGGGCGGAGGCGCGTGAGTG  GGGGAGCGCGTGGGAGTGGGGGCGGGGTGGGGCAGTCGGGGGTGG  GGAACCCGCACCCCAAACCTCTGCCAGTAGAGAACCCGAACCGGGAA  ATGGGTGCCTCAAGAAGGCCGTGGCCCTTGCACGTGGCCGGCGAGGC  AGCGCGTCCTCGGGGCCCACCCCCACCTTGCCGCCCTTGGCGTCCCGC  GCCTGCCTCTCTGTGTAGGCCGCGCCACTGCCAGGCCGCCTCTCCC  AACTCTGTGGGCCA**TGGCTGTGGTGCGTCCAGGCAGG** |
| DDX4 | 1. cg04463551 (GRCH38:Chr5:55737764) 2. cg02899723   (GRCH38:Chr5:55737786)   1. cg15190754 (GRCH38:Chr5:55737867) 2. cg10585263 (GRCH38:Chr5:55737887) 3. cg22696214 (GRCH38:Chr5:55737925) 4. cg27311866   (GRCH38:Chr5:55738049) | FP: GATGGGTAAAAGGGGAGAGAGTAT  RP: CTCCGCGACTTACTCTCCCAAA  Product length: 475 | GRCH38: chr5:55737625-55738099  **GATGGGTAAAAGGGGAGAGAGCAC**TAAAGAGGCCAAGTAGGGAGGAC  ATTATCCCTTTCCCCTTTTATTATTATTTTTTGTTAGAAAAAGGCAGGTGGA  CATATTTAGCTTGCGAAGCTTGCACAAGGAAAATCCAGAGCGTCTCCAGA  ATAAGGATCCCACGAGAGAACGTGGACACAAGTAGGCCCTCAGCCAGCC  TTGTACCCACAGGCCCAACAGGCCACTTGGCTATGAGGCCAGAGCGTCG  CCATAGGGGCCCGAACGCTAGCGTTTAGGGAATCCGCAGGCTAGAAGTG  GAGGCGGGACGCCACTGGTCGTCTGAGCGCTGATTGGCTGGTGGCGCTA  GTCACCAGCCAATCGTCAACAGACGCCATTTGTTGTTGGAGCCACGCACC  TGACGCAGTGGGCGTCTTGCACGTGCAGCCGTTTAAGTCGCGTGGGCGC  CTGCGAGGG**CTTGGGAGAGCAAGCCGCGGAG** |
| ADAD1 | 1. cg10668096 (GRCH38:Chr4:122379253) 2. cg02985694 (GRCH38:Chr4:122379301) 3. cg08374687 (GRCH38:Chr4:122379503) 4. cg09682129 (GRCH38:Chr4:122379633) | FP: GGATAGTAAGGGAGGAGGTTGAA  RP: ACCCACCAAAATTATCCTTCCT  Product length: 464 | GRCH38: chr4:122379227-122379690  **GGACAGTAAGGGAGGAGGCTGAA**CTGCGCGATTTTACCTGGCTTCTCCAG  AAGGGTAAGGCGGCCAGTTGGACCCGGTCCTTGTGTTCGGAGAACAGAGT  CACCCAGGCCTCGAACGCCTGCGATGGTCGGCGTCTCTTCCCTAGGTGACG  CAAGACGCGGAGCTCGGCTGCACGACGCTGGCGCAAGCGCGGGGGCAAG  AGCGCCGGCCTCCGAGACGGTTAGTGATTGGACGAAGCAGGGCGCGGGG  GCGCAAGCCCGGGTCCTGCAGGGGCGACGCGAGGCCTCTTTTGAAAGATG  CGGCCCTGACCCTGTGAACCTCGCGCAGAGCGGCCTGAAGCGAGAGGTTG  AGGCTGGGAGGTGGGAGCAACGGCGGCGGCGGCCGCCTGCGAGCCCCCG  GCCTGAGGCGCAGCAGCAGCTGCCCGTCTTCCCACATAGAGGGC**AGGAAG**  **GACAACTTTGGTGGGT** |
| STRA8 | 1. cg00733190 (GRCH38:Chr7:135233767) 2. cg12771165 (GRCH38:Chr7:135233901) | FP: ATTTTGCGAGGTGAGTTAGT  RP: TCACCTATTAAACTCCGCTACAC  Product length: 393 | GRCH38: chr7: 135233612-135234004  **ACCCTGCGAGGTGAGTCAGC**CCCGCCCCTCCCTCTTCTCTCTCCTCCCTTTTC  TGTCGCCTTCGGACAACTTGTAACACTGTTTCTTCCACTGCCCACACGCCCAT  TGGCCCGTCACCACGCATCCCCATTGGCTGTGGCGGCCAGGGACAGGGCCG  CGATTGGTCCCCACCCCTGTAACGAGGTGCCAGGTCTGTTTTCTGACCCAGA  CAGGAACCGCGATCCCCACTCCGGCGCACGAAGCCGGGTGACTGCTGTCCC  GGGAGTGGGGACGTCGCGTGCACCGTTGGCGAGTAAGTATCCTTTGAACGC  TCCTCTCCAGAAAGGTGCCCTTGGGCATATGAGTGTTTGTGTAGACTTGAAA  GAATTCA**GTGCAGCGGAGCCTAACAGGTGA** |
