# Supplementary material for: Tackling somatic DNA contamination in sperm epigenetic studies
Source: Front Reprod Health. 2025 Feb 5;7:1506117. doi: 10.3389/frph.2025.1506117 (PMC11835817; doi:10.3389/frph.2025.1506117)
Supplement: Supplementary file 3 [file Table2.docx]

**Supplementary Table S2: CpG sites primers detail for use as biomarkers to identify somatic cell contamination by PCR**

| **IDs** | **Coordinate** | **Primer details** | **Original sequence** | **Bisulfite converted Product sequence for methylated-specific primer** | **Bisulfite converted Product sequence for unmethylated-specific primer** |
| --- | --- | --- | --- | --- | --- |
| cg09439260  (DAZL) | GRCH38: chr3: 16604277 | MFP: TTAGAGTTGTATTTTGTGGTGGCG  MRP: CTTACTAATAACGACTCCCTCGTAT  Product length: 255  UFP: TTAGAGTTGTATTTTGTGGTGGTG  URP: CTTACTAATAACAACTCCCTCATAT  Product length: 255 | GRCH38: chr3:16604255-16604509  TCAGAGCTGCATCTTGTGGTGGCGCTGAAGCAACGCTAGGCATTTACCAAACTCACAAAAACGCACACCCAACGCTATCTACCAAATGGACATTTTGTCAAAGGATCCTGGTTTATCGAGAGGGAAAAAACCGTACCCAGAATTTAAAAATTCTAAAATTCTTAATGGTAACTGCTGAAACCAACCTCGTGCGGCAAAGATGGCGTCAGGCGAGCTTTTCTGTCGCCGCCACACGAGGGAGCCGCCATCAGCAAG | GRCH38: chr3:16604255-16604509  **TTAGAGTTGTATTTTGTGGTGGCG**TTGAAGTAACGTTAGGTATTTATTAAATTTATAAAAACGTATATTTAACGTTATTTATTAAATGGATATTTTGTTAAAGGATTTTGGTTTATCGAGAGGGAAAAAATCGTATTTAGAATTTAAAAATTTTAAAATTTTTAATGGTAATTGTTGAAATTAATTTCGTGCGGTAAAGATGGCGTTAGGCGAGTTTTTTTGTCGTCGTT**ATACGAGGGAGTCGTTATTAGTAAG** | GRCH38: chr3:16604255-16604509  **TTAGAGTTGTATTTTGTGGTG**GTGTTGAAGTAATGTTAGGTATTTATTAAATTTATAAAAATGTATATTTAATGTTATTTATTAAATGGATATTTTGTTAAAGGATTTTGGTTTATTGAGAGGGAAAAAATTGTATTTAGAATTTAAAAATTTTAAAATTTTTAATGGTAATTGTTGAAATTAATTTTGTGTGGTAAAGATGGTGTTAGGTGAGTTTTTTTGTTGTTGTT**ATATGAGGGAGTTGTTATTAGTAAG** |
| cg15180637  (DAZL) | GRCH38: chr3:  16604551 | MFP: GCGAGGGGATTAGAGGTATTTTCG  MRP: TCTCCCCGTAACTCCGCAAAATA  Product length: 219  UFP: GTGAGGGGATTAGAGGTATTTTTG  URP: TCTCCCCATAACTCCACAAAATA  Product length: 219 | GRCH38: chr3:16604529-16604747  GCGAGGGGACCAGAGGCACTTCCGGCCCAGCCCCCTCAGCTACAGGGCCATGCCTTCAGGACGCCCCACACCCCACGCTGAGGCCCCCACGAACCCCGCCCACCCCACCAAGTACAGGGA  CCAGGAGGGAACCACTTCCTAAGGAAGCTCCGGCCCTCGAAGTTTAAGAAGGCAAGTCCCTCAGCAGGCCCGCCGCCATCTTGCGGAGCCACGGGGAGA | GRCH38: chr3:16604529- 16604747  **GCGAGGGGATTAGAGGTATTTTCG**GTTTAGTTTTTTTAGTTATAGGGTTATGTTTTTAGGACGTTTTATATTTTACGTTGAGGTTTTTACGAATTTCGTTTATTTTATTAAGTATAGGGATTAGGAGGGAATTATTTTTTAAGGAAGTTTCGGTTTTCGAAGTTTAAGAAGGTAAGTTTTTTAGTAGGTTCGTCGT**TATTTT** **GCGGAGTTAC** **GGGGAGA** | GRCH38: chr3:16604529-16604747  **GTGAGGGGATTAGAGGTATTTTTG**GTTTAGTTTTTTTAGTTATAGGGTTATGTTTTTAGGATGTTTTATATTTTATGTTGAGGTTTTTATGAATTTTGTTTATTTTATTAAGTATAGGGATTAGGAGGGAATTATTTTTTAAGGAAGTTTTGGTTTTTGAAGTTTAAGAAGGTAAGTTTTTTAGTAGGTTTGTTGT**TATTTTGTGGAGTTATGGGGAGA** |
| cg22703164  (DAZL) | GRCH38: chr3:  16606101 | MFP: TTTAGGTTTTATAGGAAGGCG  MRP: CTCACGTTATAAAAATCCACCGT  Product length:264  UFP: TTTAGGTTTTATAGGAAGGTG  URP: CTCACATTATAAAAATCCACCAT  Product length:264 | GRCH38:3:16606082-16606345  TCCAGGCCTTACAGGAAGGCGCTAGGCCTGCCTAGAGAATTCTAGAACATTCTTAAGGTCAGAATACGGGCGTAGAGCAGGAGCAAGCTGTGGGCCAGTTGGTGGAGCCCATCACGTAAC  TTCGGGAACCACTGCAGCACCTTAGCTATACACACAGGGCTAAGTAGCTATGACCTTTATCCACAAACTTCACTTCTGTACTGACGGAAAGCATTAAAATGTTACTTTTTGAGCTGGCAAAACGGTGGACTTCTATAACGTGAG | GRCH38: chr3:16606082- 16606345  **TTTAGGTTTTATAGGAAGGCG**TTAGGTTTGTTTAGAGAATTTTAGAATATTTTTAAGGTTAGAATACGGGCGTAGAGTAGGAGTAAGTTGTGGGTTAGTTGGTGGAGTTTATTACGTAATTTCGGGAATTATTGTAGTATTTTAGTTATATATATAGGGTTAAGTAGTTATGATTTTTATTTATAAATTTTATTTTTGT ATTGACGGAAAGTATTAAAATGTTATTTTTTGAGTTGGTAAA**ACGGTGGATTTTTATAACGTGAG** | GRCH38: chr3:16606082-16606345    **TTTAGGTTTTATAGGAAGGTG**TTAGGTTTGTTTAGAGAATTTTAGAATATTTTTAAGGTTAGAATATGGGTGTAGAGTAGGAGTAAGTTGTGGGTTAGTTGGTGGAGTTTATTATGTAATTTTGGGAATTATTGTAGTATTTTAGTTATATATATAGGGTTAAGTAGTTATGATTTTTATTTATAAATTTTATTTTTGTATTGATGGAAAGTATTAAAATGTTATTTTTTGAGTTGGTAAA**ATGGTGGATTTTTATAATGTGAG** |
| cg10668096  (ADAD1) | GRCH38: chr4:  122379253 | MFP: AGGGAGGAGGTTGAATTGCG  MRP: CCCTACAAAACCCGAACTTAC  Product length: 264  UFP: AGGGAGGAGGTTGAATTGTG  URP: CCCTACAAAACCCAAACTTAC  Product length:264 | GRCH38: chr4:122379235-122379498  AGGGAGGAGGCTGAACTGCGCGATTTTACCTGGCTTCTCCAGAAGGGTAAGGCGGCCAGTTGGACCCGGTCCTTGTGTTCGGAGAACAGAGTCACCCAGGCCTCGAACGCCTGCGATGGTCGGCGTCTCTTCCCTAGGTGACGCAAGACGCGGAGCTCGGCTGCACGACGCTGGCGCAAGCGCGGGGGCAAGAGCGCCGGCCTCCGAGACGGTTAGTGATTGGACGAAGCAGGGCGCGGG  GGCGCAAGCCCGGGTCCTGCAGGG | GRCH38:Chr4:122379235-122379498  AGGGAGGAGGTTGAATTGCGCGATTTTATTTGGTTTTTTTAGAAGGGTAAGGCGGTTAGTTGGATTCGGTTTTTGTGTTCGGAGAATAGAGTTATTTAGGTTTCGAACGTTTGCGATGGTCGGCGTTTTTTTTTTAGGTGACGTAAGACGCGGAGTTCGGTTGTACGACGTTGGCGTAAGCGCGGGGGTAAGAGCGTCGGTTTTCGAGACGGTTAGTGATTGGACGAAGTAGGGCGCGGGGGCGTAAGTTCGGGTTTTGTAGGG | GRCH38:Chr4:122379235-122379498  **AGGGAGGAGGTTGAATTGTG**TGATTTTAT  TTGGTTTTTTTAGAAGGGTAAGGTGGTTAGTTGGATTTGGTTTTTGTGTTTGGAGAATAGAGTTATTTAGGTTTTGAATGTTTGTGATGGTTGGTGTTTTTTTTTTAGGTGATGTAAGATGTGGAGTTTGGTTGTATGATGTTGGTGTAAGTGTGGGGGTAAGAGTGTTGGTTTTTGAGATGGTTAGTGATTGGATGAAGTAGGGTGTGGGGGT**GTAAGTTTGGGTTTTGTAGGG** |
| cg00733190  (STRA8) | GRCH38: chr7:  135233767 | MFP: GCGGTTAGGGATAGGGTCG  MRP: TCACCTATTAAACTCCGCTACAC  Product length: 255  UFP: GTGGTTAGGGATAGGGTTG  URP: TCACCTATTAAACTCCACTACAC  Product length: 255 | GRCH38: chr7:135233750-135234004  GCGGCCAGGGACAGGGCCGCGATTGGTCCCCACCCCTGTAACGAGGTGCCAGGTCTGTTTTCTGACCCAGACAGGAACCGCGATCCCCACTCCGGCGCACGAAGCCGGGTGACTGCTGTC  CCGGGAGTGGGGACGTCGCGTGCACCGTTGGCGAGTAAGTATCCTTTGAACGCTCCTCTCCAGAAAGGTGCCCTTGGGCATATGAGTGTTTGTGTAGACTTGAAAGAATTCAGTGCAGCGGAGCCTAACAGGTGA | GRCH38: chr7:135233750-135234004  GCGGTTAGGGATAGGGTCGCGATTGGTTTTTATTTTTGTAACGAGGTGTTAGGTTTGTTTTTTGATTTAGATAGGAATCGCGATTTTTATTTCGGCGTACGAAGTCGGGTGATTGTTGTTTCGGGAGTGGGGACGTCGCGTGTATCGTTGGCGAGTAAGTATTTTTTGAACGTTTTTTTTTAGAAAGGTGTTTTTGGGTATATGAGTGTTTGTGTAGATTTGAAAGAATTTAGTGTAGCGGAGTTTAATAGGTGA | GRCH38: chr7:135233750-135234004  **GTGGTTAGGGATAGGGTTG**TGATTGGTTTTTATTTTTGTAATGAGGTGTTAGGTTTGTTTTTTGATTTAGATAGGAATTGTGATTTTTATTTTGGTGTATGAAGTTGGGTGATTGTTGTTTTGGGAGTGGGGATGTTGTGTGTATTGTTGGTGAGTAAGTATTTTTTGAATGTTTTTTTTTAGAAAGGTGTTTTTGGGTATATGAGTGTTTGTGTAGATTTGAAAGAATTTA**GTGTAGTGGAGTTTAATAGGTGA** |
